# Correlation between antibiotic use and changes in susceptibility patterns of *Pseudomonas aeruginosa* in a medical-surgical intensive care unit

**DOI:** 10.4103/0972-5229.40945

**Published:** 2008

**Authors:** Hatem Kallel, Fouzia Mahjoubi, Hassen Dammak, Mabrouk Bahloul, Chokri Ben Hamida, Hedi Chelly, Noureddine Rekik, Adnéne Hammami, Mounir Bouaziz

**Affiliations:** **From:** Service de Réanimation Polyvalente, CHU Habib Bourguiba, route el Ain Km 1, 3029 Sfax - Tunisia; 1 Laboratoire de microbiologie, CHU Habib Bourguiba, route el Ain Km 1, 3029 Sfax - Tunisia

**Keywords:** Ciprofloxacin, use, imipenem, *P. aeruginosa*, resistance

## Abstract

**Context:**

Multiple surveillance programmes have reported a decline in antibiotic susceptibility of *P. aeruginosa.*

**Aim:**

Our study aimed to study the relationship between the use of antipseudomonal drugs and the development of resistance of *P. aerogenosa* to these drugs.

**Setting and Design:**

Our study is retrospective. It was conducted in a medical surgical intensive care unit during a five-year period (January 1^st^, 1999 to December 31, 2003), which was divided into 20 quarters. We had monitored the use of antipseudomonal agents and the resistance rates of *P. aeruginosa* to these drugs.

**Statistical Methods:**

The associations between use and resistance were quantified using non-partial and partial correlation coefficients according to Pearson and Spearman.

**Results:**

Over the study period, the most frequently used antipseudomonal agent was Imipenem (152 ± 46 DDD/1000 patients-day) and the resistance rate of *P. aeruginosa* to Imipenem was 44.3 ± 9.5% (range, 30 and 60%). In addition, Imipenem use correlated significantly with development of resistance to Imipenem in the same (*P* < 0.05) and in the following quarter (*P* < 0.05); and Ciprofloxacin use correlated significantly with resistance to Ciprofloxacin in the following quarter (*P* < 0.05). However, use of Ceftazidime or Amikacine had no apparent association with development of resistance.

**Conclusion:**

We conclude that the extensive use of imipenem or ciprofloxacin in intensive care units may lead to the emergence of imipenem- and ciprofloxacin-resistant strains of *P. aeruginosa* and that antibiotic prescription policy has a significant impact on *P. aeruginosa* resistance rates in an intensive care unit.

## Introduction

Multiple surveillance programmes have reported *P. aeruginosa* as one of the leading causes of nosocomial infection.[1–3] In our hospital, it represents 19% of micoorganisms causing nosocomial infections[4] and in our intensive care unit, it represents 44.7% of pathogens responsible for ICU acquired infections (unpublished data). This frequency had lead to a large use of antipseudomonal agents and concomitantly to a decline in antibiotic susceptibility of *P. aeruginosa* because of its ability to acquire resistance.[5–7] Indeed, many studies had reported the influence of previous exposure to antibiotic therapy on the susceptibility pattern of *P. aeruginosa*.[7–11] This impact was called “collateral damage” from antibiotic prescription to refer to ecological adverse effects of antibiotic consumption which are represented by the emergence of multi-drug resistant organisms via selection or mutation.[12]

Because of the increasing frequency of isolation of *P. aeruginosa* and the emergence of multi-drug resistant strains in our unit, we had undertaken this epidemiological study in order to study the relationship between the use of antipseudomonal agents and the development of resistance to these drugs.

## Materials and Methods

This study was conducted at the medical surgical intensive care unit of the Habib Bourguiba University Hospital (Sfax-Tunisia). Our unit is a 22-bed intensive care unit in a 510-bed tertiary-care teaching hospital that serves as first line medical center for an urban population of one million inhabitants and as a referral center for a larger population coming from south Tunisia.

This study is a retrospective analysis of data collected prospectively. It was conducted over a five year period (January 1^st^, 1999 to December 31, 2003) which was divided into 20 quarters.

### Antimicrobial usage

Antipseudomonal agents available in our hospital are imipenem, ceftazidime, amikacine, and ciprofloxacin. Antibiotic utilization data were extracted on a quarterly basis from the inpatient pharmacy computer system and stored in a spreadsheet program (Excel®). Usage data was expressed as total grams of antibiotic dispensed per quarter and then converted to daily doses dispensed (DDD) by using the daily doses most frequently prescribed in our unit, which were as follows: imipenem, 2 g; ceftazidime, 3 g; amikacine, 1 g; intravenous ciprofloxacin, 0.4 g; oral ciprofloxacin, 1g.

### Microbiology and susceptibility data

*P. aeruginosa* was identified in the laboratory by using standard clinical microbiology methods.[13] Antimicrobial susceptibility was determined by disk diffusion methods according to the recommendations of the National Committee for Clinical Laboratory Standards (NCCLS).[14] An isolate was considered susceptible, intermediate, or resistant according to the criteria of the NCCLS. The isolates with intermediate susceptibility were classified as resistant for analysis.

Susceptibility data for *P. aeruginosa* were obtained quarterly using a computer based documentation system. The system is adjusted to count not only primary isolates from individual patients, but also to include follow-up isolates if the primary isolates show a different pattern of antibiotic resistance. Duplicate isolates, defined as the same bacterial species from the same patient with the same antibiogram, were removed.

### Data analysis

Categorical variables were expressed in percentage and continuous variables in means (±SD). Relationships between increasing antibiotic use and the resistance rates of *P. aeruginosa* were analyzed to determine the likelihood of a correlation between antibiotic utilization and the emergence of resistance. A linear curve regression was performed on relevant variables and the associations of primary interest from the correlation analysis were tabulated, showing correlation coefficient (r^2^) and significance (*P*). Statistical significance was defined as a *P*-value equal or less than 0.05 for the corresponding correlation coefficient (r^2^). In addition, the associations between consumption and resistance to ceftazidime, imipenem, amikacine and ciprofloxacin were quantified using non-partial and partial correlation coefficients according to Pearson and Spearman.

## Results

Over the study period, the mean (±SD) number of patients hospitalized in our unit was 299 ± 20 hospitalizations per quarter (range: 267 and 339 hospitalizations per quarter). The mean number of hospitalization day was 1766 ± 250 hospitalization day per quarter (range, 1374 and 2358 hospitalization day per quarter) and the mean occupation rate in the unit was 88 ± 13% (range, 69 and 119%).

**Figure 1:**
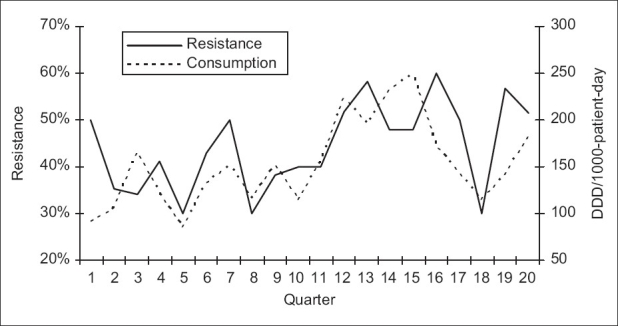
Correlation between consumption of imipenem and resistance of *P. aeruginosa* to imipenem: quarterly resistance rates plotted against quarterly consumption rates during the 20 quarters of the study

**Figure 2:**
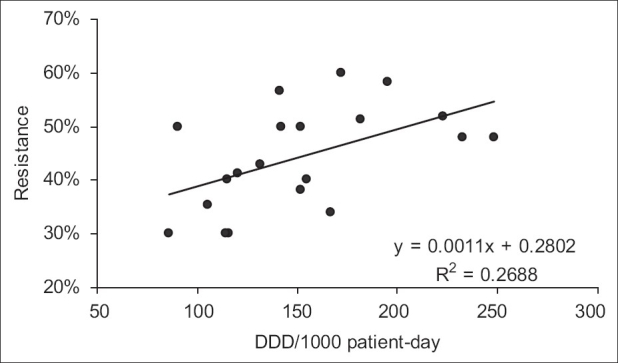
Linear regression showing the statistically significant association between quarterly imipenem consumption and resistance of P. aeruginosa to imipenem in the same quarter during the 20 quarters of the study (*P* < 0.05)

**Figure 3:**
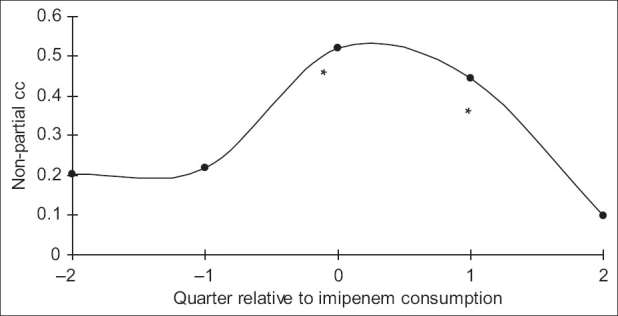
Non-partial correlation coefficients between quarterly imipenem consumption and resistance in the quarter of consumption (designated “0”) and the 2 quarters prior to and following consumption. Asterisks indicate statistical significance (*P*<0.05)

**Figure 4:**
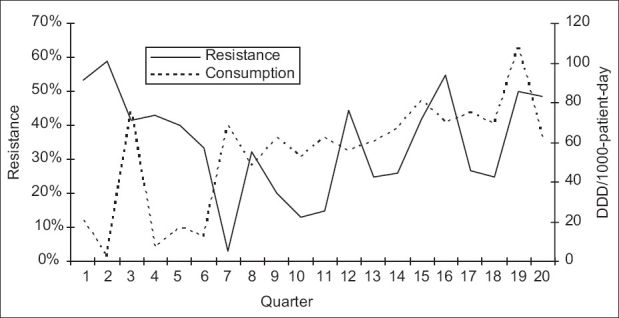
Correlation between consumption of ceftazidime and resistance of *P. aeruginosa* to ceftazidime: quarterly resistance rates plotted against quarterly consumption rates during the 20 quarters of the study

**Figure 5:**
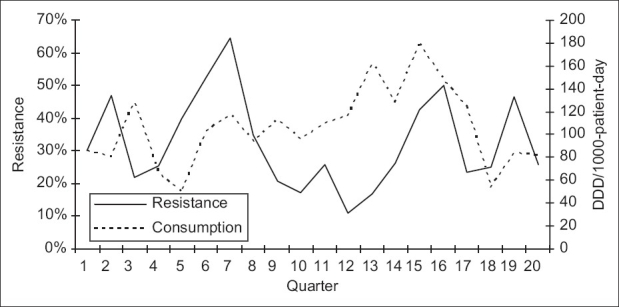
Correlation between consumption of amikacine and resistance of *P. aeruginosa* to amikacin: quarterly resistance rates plotted against quarterly consumption rates during the 20 quarters of the study

**Figure 6:**
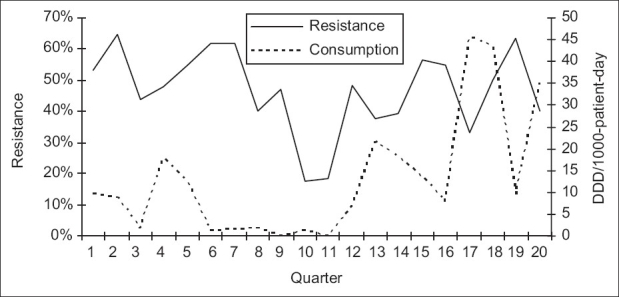
Correlation between consumption of ciprofloxacine and resistance of *P. aeruginosa* to ciprofloxacine: quarterly resistance rates plotted against quarterly consumption rates during the 20 quarters of the study

**Figure 7:**
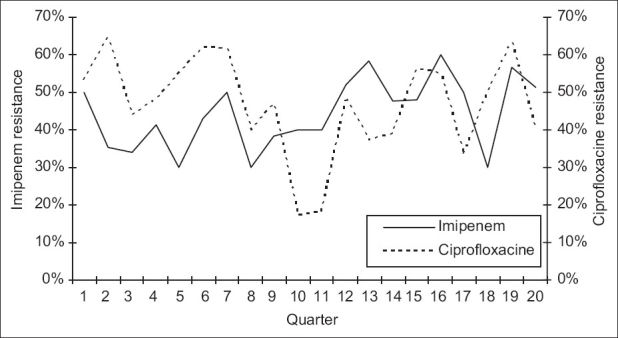
Correlation between resistance of *P. aeruginosa* to imipenem and to ciprofloxacine: quarterly resistance rates of *P. aeruginosa* to imipenem plotted against quarterly resistance rates to ciprofloxacine during the 20 quarters of the study

**Figure 8:**
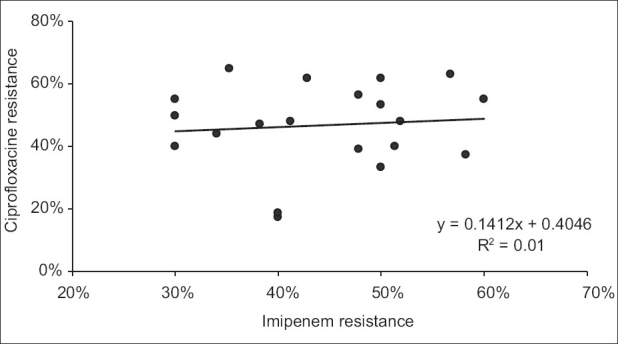
Linear regression showing no statistically significant association between quarterly resistance of *P. aeruginosa* to imipenem and quarterly resistance of *P.aeruginosa* to ciprofloxacine (*P* > 0.05)

Over the study period, 583 *P. aeruginosa* isolates were studied (29 ± 10 isolates per quarter). Three hundred and eighty-seven of them (66.4%) were isolated from pulmonary samples, 110 (18.9%) from blood samples and 86 (14.7%) from urinary samples. The resistance rate of *P. aeruginosa* to imipenem was 44.3 ± 9.5% (range, 30 and 60%). The most frequently used antipseudomonal agents were imipenem (152 ± 46 DDD/1000 patients-day) and amikacine (106 ± 34 DDD/1000 patients-day). Over the study period, imipenem use correlated significantly with imipenem resistance (r^2^ = 0.26, *P* < 0.05) [Figures 1 and 2]. This correlation was seen not only when quarterly prescription rates were compared with resistance data from the same quarter, but also when compared with those of the following quarter [Figure 3]. In addition, ciprofloxacin use correlated significantly with resistance to ciprofloxacin observed in the following quarter. However, no apparent association was found between use and resistance for ceftazidime (r^2^ = 0.045, *P* > 0.1) nor for amikacine (r^2^ = 0.000, *P* > 0.1). Table 1, [Figures 4–6]. In addition, resistance of *P. aeruginosa* to imipenem does not correlate with its resistance to ciprofloxacin (r^2^ = 0.01, *P* > 0.1) [Figures 7 and 8].

**Table 1:** Partlal coefficient of correlation between antibiotic consumption and resistance of ***P. aeruginosa.*** Coefficients for the quarter of antibiotic consumption and quarter following and before consumption are given. Boldface indicates significance (***P*** ≤ 0.005)

	**Last quarter**	**Same quarter**	**Next quarter**
Ceftazidime	0.039	−0.9	0.308
Imipenem	0.22	0.52	0.443
Amikacin	−0.002	−0.131	−0.17
Ciprofloxacine	−0.04	−0.98	0.473

## Discussion

Our study shows the high level of resistance of *P. aeruginosa* against ceftazidime, amikacine, imipenem and ciprofloxacin in our unit. In addition, it shows the high level of use of antipseudomonal agents and confirms the correlation between the evolution of resistance to imipenem or ciprofloxacine and that of their consumption.

Different studies had reported *P. aeruginosa* as one of the most frequently isolated microorganisms in intensive care unit[2,3,15] and emphasized its ability to acquire resistance toward antipseudomonal agents mainly to imipenem.[8,9,16,17] Indeed, the resistance rate of *P. aeruginosa* to imipenem is increasing and can reach 24% in certain institutions[18] rekindling interest in polymixins as a last resort in the treatment of nosocomial infections caused by multidrug resistant *P. aeruginosa*.[19,20]

The multidrug resistance of *P. aeruginosa* had been correlated to prior exposure to antibiotics mainly to β-lactams.[7–9,11,17,21] Indeed, Loeffler *et al*,[16] found a correlation between the resistance of *P. aeruginosa* to piperacillin and the consumption of piperacillin (r = 0.73; *P* < 0.005) or that of piperacillin-tazobactam (r = 0.61; *P* < 0.05), between the resistance to ceftazidime and the consumption of cephalosporins (r = 0.79; *P* < 0.001), between the resistance to gentamicin and the consumption of gentamicin (r = 0.64; *P* < 0.05) or that of aminoglycosides (r = 0.76; *P* < 0.005). Lepper *et al*,[9] found a correlation between the consumption of imipenem and the resistance of *P. aeruginosa* to imipenem, to ceftazidime and to piperacillin-tazobactam. This association existed between the consumption and the resistance during the same month and during the following month. Moreover, Mutnick *et al*,[21] reported a correlation between the use of meropenem (r = 0.98), ciprofloxacine (r = 0.92) and ceftazidime (r = 0.83) and the resistance of *P. aeruginosa* toward these antibiotics. Carmeli *et al*,[11] in a retrospective study demonstrated that the consumption of imipenem was the independent factor related to the development of resistance of *P. aeruginosa* (OR = 2.8; IC_95%_ = 1.2-6.6; *P* = 0.02) toward piperacillin, imipenem or ciprofloxacine. In a case-control study, Paramythiotou *et al*,[8] demonstrated that the resistance of *P. aeruginosa* to ceftazidime was correlated to the previous consumption of piperacillin or of ticarcillin (*P* = 0.01) and that the resistance to imipenem was correlated to the previous consumption of imipenem (*P* = 0.01). El Amari *et al*,[7] in a retrospective study had looked for the factors correlated with the resistance of *P. aeruginosa.* Using multivariate analysis, they found that the exposure to any antipseudomonal antibiotic as a monotherapy was associated with an increased risk of subsequent resistance to itself (*P* = 0.006; OR = 2.5; IC_95%_ = 1.3-4.8). Troillet *et al*,[17] demonstrated that a previous exposure to imipenem was statistically correlated to the resistance of *P. aeruginosa* to imipenem (*P* = 0.0004; OR: 23.2; IC _95%_: 4.1-132.7). All these correlations translate the impact of antibiotic prescription on ecology. In addition, they demonstrate that the resistance of *P. aeruginosa* to antibiotics mainly to imipenem is associated with previous exposure to the antibiotic under question and that the exposure to an antipseudomonal agent as a monotherapy can lead to a great risk of development of resistance against this drug.

In our study, we found a statistically significant relationship between the use of imipenem and the resistance of *P. aeruginosa* to imipenem in the same and in the following quarter; and a statistically significant relationship between the consumption of ciprofloxacin and the resistance of *P. aeruginosa* to ciprofloxacine in the following quarter. This correlation is consistent with many other studies where resistance to imipenem or ciprofloxacin was found to correlate with their previous use. This consideration justifies the large effort provided by intensivists to avoid the misusage of antibiotics. Indeed, in many studies the antibiotic prescription was found to be inadequate or abusive in a large part of the cases.[22]

There are three types of epidemiological studies which can potentially link the antibiotic use with the ecological adverse effects.[12] The first type is case-control studies,[8,11,17] the second type of study assesses accumulated data on antibiotic use and correlates them with rates of antibiotic resistance[9,10] and the third type assesses an intervention aimed at limiting the use of an antibiotic to decrease the resistance to this antibiotic.[9] Our study's design corresponds to the second type of studies. It analyzes the evolution of antibiotic use and the emergence of resistance in the unit. It provides information about the impact of the overuse of antipseudomonal agents and the beneficial effect of their restriction on the ecology of an intensive care unit.

## Conclusion

Our data support that the large use of imipenem or ciprofloxacin in intensive care unit may lead to the emergence of imipenem-resistant or ciprofloxacin-resistant strains of *P. aeruginosa*. Thus, they support the concept that antibiotic prescription policy of an intensive care unit has a significant impact on bacterial resistance rates.
